# Is *Helicobacter pylori* Infection the Primary Cause of Duodenal Ulceration or a Secondary Factor? A Review of the Evidence

**DOI:** 10.1155/2013/425840

**Published:** 2013-03-27

**Authors:** Vikram Kate, N. Ananthakrishnan, Frank I. Tovey

**Affiliations:** ^1^Department of General and Gastrointestinal Surgery, Jawaharlal Institute of Postgraduate Medical Education and Research, Pondicherry 605006, India; ^2^Mahatma Gandhi Medical College & Research Institute, Pondicherry 607402, India; ^3^Division of Surgery and Interventional Science, University College London, London W1W 7ET, UK

## Abstract

*Helicobacter pylori* (*H. pylori*) has a role in the multifactorial etiology of peptic ulcer disease. A link between *H. pylori* infection and duodenal ulcer disease is now established. Other contributing factors and their interaction with the organism may initiate the ulcerative process. The fact that eradication of *H. pylori* infection leads to a long-term cure in the majority of duodenal ulcer patients and the fact that the prevalence of infection is higher in ulcer patients than in the normal population are cogent arguments in favor of it being the primary cause of the ulceration. Against this concept there are issues that need explanation such as the reason why only a minority of infected persons develop duodenal ulceration when infection with *H. pylori* is widespread. There is evidence that *H. pylori* infection has been prevalent for several centuries, yet duodenal ulceration became common at the beginning of the twentieth century. The prevalence of duodenal ulceration is not higher in countries with a high prevalence of *H. pylori* infection. This paper debate puts forth the point of view of two groups of workers in this field whether *H. pylori* infection is the primary cause of duodenal ulcer disease or a secondary factor.

## 1. It Is the Primary Cause of Ulceration (N. Ananthakrishnan and Vikram Kate)

### 1.1. *Helicobacter pylori* Is the Primary Cause of Duodenal Ulcer

The isolation of *Helicobacter pylori (H. pylori)* from the gastric mucosa generated excitement when it was postulated by Marshall that these microorganisms could be the cause of gastritis and play an important role in the etiology of peptic ulcer disease [1]. *H. pylori* infection is almost always associated with an inflammatory response; however, peptic ulcer disease and gastric carcinoma occur only in a subset of individuals chronically infected with *H. pylori*. Presumably, both bacterial and host factors contribute to this differential response.

The role of *H. pylori* as a gastric pathogen is dependent on virulence factors and pathogenic mechanisms. Virulence factors are those that allow *H. pylori* to survive in the hostile environment of the gastric lumen which includes its spiral shape, motility, adaptive enzymes, proteins, and ability to adhere to gastric mucosal cells and mucus [2]. Pathogenic mechanisms are those that lead either directly to disruption of the gastric mucosal barrier including its toxins like Vac A and Cag A and mediators of inflammation.

### 1.2. Pathogenic Mechanisms and Virulence Factors

The spiral shape and flagella of the organism allow efficient motility in the mucus and in the gastric juice. The enzyme urease by breaking down urea in the gastric juice appears to generate enough bicarbonate and ammonium ions around the organism to allow its safe passage through the gastric acid barrier to reach the protective mucous layer. Ammonia elevates the pH of the gastric mucous layer from about 6 to 7 [3]. It is known to deplete aerobic cells of alpha keto-glutarate, an essential substrate for the tricarboxylic acid cycle. Ammonia in high concentration induces vacuoles exactly the same as those seen when cells are exposed to the Vac A toxin of *H. pylori* [4]. Once within the gastric mucus, *H. pylori* is able to attach itself to phospholipids such as phosphatidyl ethanolamine, sialylated glycoproteins such as ganglioside monosialic 3 (GM3), and Lewis B antigens present in persons with blood group O [5, 6]. Once attached to the mucus layer and the mucosa, *H. pylori* secretes soluble proteases and phospholipase, which may be harmful to both the integrity of the mucus layer and the underlying cells. The “wettability” of gastric mucus is increased when *H. pylori* is present perhaps due to partial lysis of the phospholipid component [7].

One of the most important aspects of *H. pylori *pathogenicity is the “vacuolating cytotoxin” which is expressed in nearly all patients with *H. pylori* associated duodenal ulcer [8, 9]. The marker for cytotoxin is a gene for the cytotoxin protein called Vac A. A second protein at 127 kDa is called cytotoxin-associated gene A or Cag A. Cag A is a marker for the vacuolating toxin effect and the gene for Cag A is only present when VacA cytotoxin effect is present. The organisms have been classified into type I organisms which have Cag A and Vac A which are more ulcerogenic and type II organisms that lack Cag A and do not produce cytotoxins [8, 9]. Antibodies to the toxin are present in nearly all duodenal ulcer patients. This is one of the factors which determine that all patients with *H. pylori* do not have duodenal ulcer disease. 

Recently, a novel virulence factor, duodenal ulcer promoting gene A (dupA), has been identified and found to be associated with disease in some populations. A recent meta-analysis investigated the relationship of dupA genotypes and *H*. *pylori*-related clinical outcomes by using previous reports of 2,358 patients from around the world with dupA-positive genotypes. It was found that in 48%, dupA was associated with duodenal ulcer (*P* = 0.001, odds ratio OR = 1.4, confidence interval CI = 1.1–1.7); however, the prevalence of dupA positivity and its association with disease differed among the various regions around the world [10]. In another study from India, a total of 140 *H. pylori* strains isolated from duodenal ulcer (*n*  =  83) and nonulcer dyspepsia (NUD) patients (*n*  =  57) were screened by PCR and dot-blot hybridization to determine the presence of the open reading frames (ORFs) “*jhp0917* and *jhp0918*” [11]. The PCR and dot-blot results indicated the presence of “*jhp0917* and *jhp0918”* in 37.3* *% (31/83) and 12.2* *% (7/57) of *H. pylori* strains isolated from duodenal ulcer and nonulcer dyspepsia patients, respectively. The prevalence of dupA was significantly greater among strains isolated from patients with duodenal ulcer than from patients with NUD in this population (*P*  =  0.001, odds ratio  =  4.26, confidence interval* * = * *1.60–11.74). The authors suggested that dupA can be considered a biomarker for duodenal ulcer patients in India.

Levi et al. reported increased gastrin levels due to *H. pylori* infection which induced increased gastric acid secretion leading to duodenal ulcer [12]. Eradication of *H. pylori* abolishes the hypergastrinemia suggesting that this is due to *H. pylori* infection.

Somatostatin deficiency is seen in the gastric antrum in patients infected with *H. pylori* [13]. Subsequently, it was discovered that immunoreactive somatostatin, D cells, and somatostatin message were all decreased in patients with gastritis [13, 14]. As a result of either genetic predisposition or an alteration in G-cell or D-cell function due to *H. pylori* infection, some patients will develop an increased parietal mass. The increased parietal mass results in an increased acid load that leads, in some patients, to gastric metaplasia in the duodenum. *H. pylori*-associated antral gastritis appears to be prerequisite for colonization of areas of duodenal metaplasia and the appearance of duodenitis and duodenal ulceration [14]. In patients with *H. pylori-* related duodenal ulcer it was found that duodenal colonization by *H. pylori* in patients with nonulcer dyspepsia is strongly predictive of the subsequent development of duodenal ulcer [15]. These factors provide a link between *H. pylori*, gastritis, acid hypersecretion, and peptic ulceration.

### 1.3. Role of *H. pylori* in Duodenal Ulcer Disease

#### 1.3.1. Uncomplicated Duodenal Ulcer

Studies have shown that *H. pylori *has a role in the multifactorial etiology of peptic ulcer disease and there is interplay of many factors such as the acid attack and the mucosal defence [12, 14, 16]. It is modulated by genetics, gender, blood group, smoking, age, and various physiologic considerations, including acid output [16]. These and other considerations explain the discrepancy between the high frequency of *H. pylori* infection in the population and a less than 10% overall lifetime prevalence of duodenal ulcer disease. 

The prevalence of *H. pylori* infection in duodenal ulcer has consistently been found to be between 90% and 100% [17, 18]. In our earlier report we found that patients with duodenal ulcer had a significantly higher prevalence of *H. pylori* at 91% compared to normal controls [19]. Most agents used for treatment of duodenal ulcer are aimed at reducing acid secretion and promote healing by minimizing acid attack; however, this antisecretory therapy has no effect on the *H. pylori* status and does not correct the underlying state of gastroduodenitis. The mucosa, therefore, remains abnormal and vulnerable to ulcer relapse following cessation of antisecretory therapy. Therefore, treatment that fails to address the role of *H. pylori* in the causation of the mucosal inflammation, which predisposes to ulceration, is likely to confer only short-term benefit.

Eradication of infection has been shown beyond doubt to markedly alter the natural history of duodenal ulcer disease. A number of series have shown either low or no recurrence of ulcer at the end of one year compared with a natural recurrence rate of more than 70% [20, 21]. Several studies, the Maastricht III Consensus Report and the Second Asia-Pacific Consensus Guidelines for *Helicobacter pylori* infection, have demonstrated that ulcers recur in only a small percentage of patients following successful *H. pylori* eradication in comparison to a recurrence rate of 50% or greater within the course of one year when the organism persists and hence *H. pylori* eradication is recommended in patients with duodenal ulcer disease [22–28]. In a Cochrane Systematic Review, it was concluded that *H. pylori* eradication therapy is effective treatment for *H. pylori* positive peptic ulcer disease [29]. However, eradication has proved to be difficult and reinfection rates are high in developing countries. Recrudescence or reinfection with *H. pylori* is common and may be an important factor in recurrence of peptic ulcer disease since successful eradication virtually abolishes recurrence of duodenal ulcer [30]. Improper selection of therapy such as nitroimidazole comprising regimens in developing countries or factors such as low compliance due to use of complex regimen lead to a low efficacy of the therapy resulting in recrudescence or reinfection. Recrudescence is more often the cause of recurrence of peptic ulcer rather than recurrence following successful eradication.

The previous reports corroborate the view that *H. pylori *infection is the most important determinant in preventing ulcer relapse in patients with duodenal ulcer and supports its role as a primary factor in duodenal ulcer disease.

#### 1.3.2. *Helicobacter pylori* and Complicated Duodenal Ulcer Disease

The major complications of duodenal ulcer disease are bleeding, perforation, and gastric outlet obstruction.

*Bleeding. *Bleeding is by far the most frequent complication of chronic duodenal ulcer disease. 30% of bleeding ulcers bleed massively [31]. Most of the studies report a high prevalence of *H. pylori* in bleeding duodenal ulcer. Kadayifei reported a higher prevalence rate of *H. pylori* infection in patients with bleeding duodenal ulcer (88%) compared to patients with uncomplicated duodenal ulcer (67.2%) [32]. He recommended eradication therapy for *H. pylori* for all patients with *H. pylori* positive duodenal ulcer to prevent recurrent bleeding. We too found a significantly higher prevalence of *H. pylori* in patients with bleeding duodenal ulcer at 89% when compared to controls at 60% [33]. Studies have shown that persistent *H. pylori* infection was an independent predictor of recurrence of duodenal ulcer bleeding [34–36]. It has also been seen that Cag A positive *H. pylori* infection is associated with an increased risk of bleeding [37].

 In another Spanish study of 103 patients with bleeding duodenal ulcer, it was found that at a median followup of 27 months there were no instances of rebleeding in any of the 93 eradicated patients suggesting that bleeding from duodenal ulcer is virtually abolished if patients receive *H. pylori* eradication therapy [38]. A recent study reported that peptic ulcer rebleeding virtually does not occur in patients with bleeding duodenal ulcers following *H. pylori* eradication [39].

From the present data available in the literature it appears that persistence of *H. pylori* infection is one of the most important factors causing rebleeding in patients with bleeding duodenal ulcer and hence eradication therapy should be recommended as a routine in all patients with bleeding duodenal ulcer positive for *H. pylori* infection.

*Gastric Outlet Obstruction*. Although the conventional surgery of vagotomy and drainage is commonly performed for this complication of duodenal ulcer, some authors have advocated endoscopic balloon dilatation and *H. pylori* eradication for this group [40, 41]. Lam et al. recommended endoscopic dilatation with *H. pylori* eradication for patients with duodenal ulcer and gastric outlet obstruction [42]. We had found a high prevalence of *H. pylori* in patients with duodenal ulcer and gastric outlet obstruction [43]. This was true whether or not the ulcer was active or cicatrized. Gisbert and Pajares recommended in a review article on *H. pylori* and gastric outlet obstruction that treatment should start pharmacologically with the eradication of *H. pylori* whereas dilatation or surgery should be reserved for patients who do not respond to medical therapy [44]. These reports suggest that although a dilatation is carried out to relieve benign gastric outlet obstruction, eradication of *H. pylori* is an important component of this therapy as obstructed ulcers are also associated with *H. pylori*.

*Perforation. *In the last few years reports are being published on the role of *H. pylori* in perforated duodenal ulcer [45–49]. Earlier reports on the association between *H. pylori* and perforated duodenal ulcer in the nineties suggested that the prevalence of *H. pylori* was high in patients with perforated duodenal ulcer [50–52]. 

With the association of *H. pylori* infection with perforated duodenal ulcer postulated by many studies, attention was focused on the effect of eradication of the organism on the ulcer recurrence following simple closure of perforated duodenal ulcer [45–49, 53, 54]. In an earlier study from our institute on a prospective group of 202 patients and a retrospective group of 60 patients who had undergone simple closure of perforated duodenal ulcer it was found that at every interval of followup *H. pylori* infection rate was significantly higher in patients who had recurrent or residual ulcer [55]. Metzger et al. suggested that an immediate and appropriate *H. pylori* eradication therapy for perforated duodenal ulcers reduces the relapse rates after simple closure [56]. In a recent study on 150 patients with perforated duodenal ulcer following simple closure included on a prospective basis, we found that presence of recurrent ulcer was 18.6% in the eradicated patients when compared to 70% in noneradicated patients suggesting that *H. pylori* eradication reduces risk of ulcer recurrence after simple closure [57].

In a recent systematic review and meta-analysis on *H. pylori* eradication therapy after simple closure of perforated duodenal ulcer, the pooled incidence of 1-year ulcer recurrence in the *H. pylori* eradication group was 5.2% (95% confidence interval (CI) of 0.7 and 9.7), when compared with that of the control group (35.2%) with 95% CI of 0.25 to 0.45 [47]. The pooled relative risk was 0.15 with 95% CI of 0.06 to 0.37. The authors concluded that *H. pylori* eradication after simple closure of duodenal ulcer perforation gives better results than antisecretory noneradication therapy for prevention of ulcer recurrence and hence should be recommended for all infected patients.

From all the previously mentioned data it appears that *H. pylori* infection does play an important role in perforated duodenal ulcer and eradication is recommended in all infected patients following simple closure to prevent ulcer relapse.

### 1.4. Conclusion

The strong evidence in the literature linking *H. pylori* etiologically to duodenal ulcer and reports on eradication therapy of *H. pylori* in preventing relapse of uncomplicated and complicated duodenal ulcer suggest that *H. pylori is the primary cause of duodenal ulcer.*

## 2. It Is Not the Primary Cause but a Secondary Factor Delaying Healing (Frank I. Tovey)

The fact that eradication of *H. pylori *infection leads to a long-term cure in the majority of duodenal ulcer patients and the fact that the prevalence of infection is higher in ulcer patients than in the normal population are cogent arguments in favor of it being the primary cause of the ulceration. Against this concept there are difficulties that need explanation.

- Infection with *H. pylori *is widespread yet only a minority of infected persons develop duodenal ulceration [58].
- There is evidence that *H. pylori *infection has been prevalent for several centuries; yet duodenal ulceration became common at the beginning of the twentieth century [59–66].
- The prevalence of duodenal ulceration is not higher in countries with a high prevalence of *H. pylori* infection as would be expected if it were causal. Geographically the prevalence of duodenal ulceration does not correspond to the prevalence of *H. pylori*. Within countries with the same overall prevalence of *H. pylori* infection the prevalence of duodenal ulceration may vary from region to region [67–81].

One explanation of these problems is that virulent strains of *H. pylori *have emerged and could account for the onset of duodenal ulceration at the turn of the twentieth century and for the geographical variations in the prevalence of ulceration. This concept is supported by the finding that duodenal ulcer patients are more likely to be infected with virulent strains than the normal population. Against this concept is the fact that the prevalence of duodenal ulceration in a country is not related to the prevalence of virulent strains. In countries where the prevalence of virulent strains is high there is no corresponding increase in the prevalence of duodenal ulceration [82].

Besides the previous difficulties there are other anomalies that cast doubt as to whether *H. pylori* infection could be the primary factor in duodenal ulceration.

- There are a large number of endoscopy reports from different countries of *H. pylori*-negative duodenal ulceration unrelated to NSAIDs ranging from 14% to 72% and occurring more often in countries with a low prevalence of *H. pylori* infection [68, 83–87] and in patients with a short history of ulceration [88, 89]
- There are several reports of duodenal ulcer recurring after eradication without reinfection [68, 84].
- As low as 50% of acute duodenal ulcer perforations are *H. pylori *negative [90].

Thus if the organism is not responsible for actually causing duodenal ulcer, we need to find another cause for the patients who have an ulcer whether they are infected or not.

### 2.1. The Role of Acid

For many years gastric acid secretion was regarded as the primary cause of duodenal ulceration. Surgical measures or medical treatment with antisecretory drugs resulted in long-term cure of the ulceration despite persistence of *H. pylori *infection. 

Patients with duodenal ulcer lie in two groups related to their maximal secretory capacity: those lying above the 95% tolerance limits of the normal population and those lying within the normal limits. In each band of secretion within the normal range, the risk of developing a duodenal ulcer increases with increasing maximal acid secretion, until at greater than the 95% upper tolerance limit of the population most developed duodenal ulceration [91]. *H. pylori *infection is thought to produce an increase in acid secretion due to hypergastrinemia resulting from colonization of the antrum. However, colonization is rarely confined to the antrum, and as a result of gastritis involving the corpus it has been shown that patients with duodenal ulcer who are infected actually have a smaller maximal gastric secretion than those who are not infected [92].

Below the lower border of the normal range in nonulcer individuals there is a band of secretion in which no subject with peptic ulceration lies, fitting in with the dictum of Schwarz, “No Acid, No Ulcer” [93].

In conclusion, in duodenal ulcer patients lying within the normal range of acid secretion some other factors in addition to acid must prevail predisposing to the ulceration and this is probably related to mucosal resistance to the effect of acid.

### 2.2. The Role of Mucosal Resistance

At first sight it seems obvious that the *H. pylori *infection must be this additional factor affecting mucosal resistance but this would not fit in with the discrepancies noted previously, in particular that a large number of people with *H. pylori *infection in the normal range of acid secretion do not develop duodenal ulcers. (An alternative role for *H. pylori* infection is described later.)

Smoking and nonsteroidal anti-inflammatory drugs (NSAIDs) are known to be such predisposing factors. A more important and universal factor is the presence or absence of protective substances in the diet which protect the mucosa. The geographical differences in the prevalence of duodenal ulceration [94–98], which are unrelated to the prevalence of *H. pylori *infection, do bear relationship to the content of protective lipids in the staple diets (certain phospholipids and sterols) [99, 100]. These could also account for the appearance of duodenal ulceration at the beginning of the twentieth century which coincided with the introduction of roller milling resulting in the increasing refinement of wheat, maize, and rice and the removal of these protective lipids.

### 2.3. The Role of *H. pylori* Infection

It is generally assumed that *H. pylori *infection is a chronic infection, but in countries with a low prevalence of *H. pylori *infection it has been shown that it can be labile, depending on the level of acid secretion in the stomach [101]. *H. pylori* colonization is very dependent on the pH levels of the acid in the stomach. In vitro, growth of *H. pylori *is restricted to pH levels of 6.5–7.5, the optimal pH being 7.0. At pH 3.0–3.5 the organism is no longer viable except in the presence of urea, as occurs in the stomach. *H. pylori* produces urease and this reacts with urea producing ammonia and growth can occur down to a pH of 1.5, below which the organism is not viable [102–104].Thus colonization of the stomach by *H. pylori* depends on favorable pH levels; both highly acid and highly alkaline conditions kill the organism, the range permitting growth in the stomach being between pH 1.5 and pH 7.5. At pH levels below 1.5 and above 7.5 it cannot survive.

The number of *H. pylori* negative duodenal ulcer patients in countries of low *H. pylori* prevalence is high and occurs predominantly in those with a short history of ulceration [88, 89]. It is probable that in these patients the initial level of acidity is high enough to cause the ulceration and also high enough to prevent *H. pylori *colonization. The presence of *H. pylori *in later cases may be the result of treatment and the reduction of acid secretion, permitting colonization with *H. pylori*. It is in this process of colonization that virulent strains may outperform nonvirulent ones, accounting for the observed preponderance of virulent strains in patients with duodenal ulcer.

It is noteworthy that when Marshall et al. in 1984 infected himself with *H. pylori,* he had taken a dose of ranitidine to reduce his acid secretion [105]. Two weeks later when the acid level had returned to normal the *H. pylori *infection had spontaneously disappeared.

The toxins released by *H. pylori* interfere with neoangiogenesis and with the healing of wounded duodenal epithelial cells [106, 107]. In patients with duodenal ulcer migration of tongues of antral mucosa secreting neutral mucus (duodenal gastric metaplasia) occurs in the duodenum, possibly related to distal displacement by an increased parietal cell mass. *H. pylori *cannot colonize normal duodenum in the presence of the acid mucin secreted by the goblet cells, but because of the neutral mucus secreted by antral mucosa *H. pylori *organisms from the stomach can colonize these areas of metaplasia. The toxic effect of this local colonization of the duodenum by *H. pylori* prevents the ulcer from following its natural course of healing and makes it chronic. Eradication of the infection permits the healing process to take place.

### 2.4. Conclusion

There is no doubt of the value of *H. pylori *eradication leading to long-term healing of duodenal ulcers, but this does not mean that the *H. pylori *infection is the initial or primary cause of the duodenal ulceration. The most important cause remains to be acid secretion, which in cases of high acid secretion may be the sole cause, but in other cases is combined with reduced mucosal resistance, in which the absence of dietary protective lipids is an important factor. 
